# Integrative Modular, Network‐Based, and Machine Learning Framework for Predicting Accessory Genome Functions and Virulence in *Escherichia coli* O157:H7

**DOI:** 10.1002/mbo3.70323

**Published:** 2026-06-10

**Authors:** Sydney Menzeko Gambushe, Oliver Tendayi Zishiri

**Affiliations:** ^1^ Discipline of Genetics, School of Agriculture and Science, College of Agriculture, Engineering and Science University of KwaZulu‐Natal Durban South Africa

**Keywords:** accessory genome, co‐occurrence network, *E. coli* O157:H7, Fisher's exact, Jaccard distance, PCA, plasmid replicons, random forest

## Abstract

The pathogenicity of *Escherichia coli* O157:H7 is shaped not only by chromosomal toxins such as *Stx* and *eae* but also by virulence and resistance genes carried on plasmids. To explore the modular structure and predictive potential of these accessory elements, presence/absence data from 77 strains (70 accessory features) were analyzed. Methods included clustering using Euclidean and Jaccard distances, gene‐to‐gene network construction with community detection, Fisher's exact tests for associations between plasmid and virulence or antimicrobial resistance genes (AMR), random forest modeling to predict virulence labels and toxin presence (excluding direct toxin markers), and PCA for visualization. Both clustering approaches revealed broad groupings, though Jaccard clustering better captured co‐occurring gene patterns. The co‐occurrence network identified 12 modules, including a prominent plasmid–virulence module centered on *IncF* replicons, *stx2*, *ehxA*, *toxB*, and *espP*. Fisher's tests showed significant associations, notably between *IncFIA* and *stx2c* (*p* = 5.3 × 10^−4^). The Random Forest classifier achieved a cross‐validated AUC of 0.853 ± 0.067, with *gad*, *espF*, *IncFIA*, and *ehxA* as key predictors. PCA explained 31.1%, 12.9%, and 10.0% of the variance across the first three components, separating plasmid–virulence module carriers from others. These findings indicate that a modular accessory genome structure contributes to the diversity of O157:H7. *IncF* plasmids and associated effectors form a highly interconnected subnetwork, and accessory markers independent of direct toxin genes can effectively predict virulence status, offering potential for rapid genomic surveillance of Shiga toxin‐producing *E. coli* (STEC).

## Graphical abstract

An analysis of the accessory genomes from 77 *Escherichia coli* O157:H7 strains using network and machine learning techniques uncovers modular communities of plasmid‐related virulence associated with *IncF* replicons. Accessory markers that are not directly linked to toxin genes can effectively predict the virulence status with an AUC of 0.85, facilitating rapid genomic monitoring of the pathogenic potential of STEC.

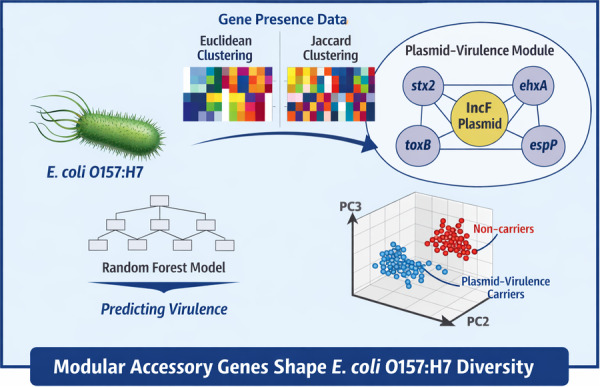

## 1 Introduction

*Escherichia coli* (*E. coli*) O157:H7 is a pathogen associated with foodborne illnesses, including hemorrhagic colitis and hemolytic‐uremic syndrome (Kotloff et al. 2013; Lozano et al. 2012). Its ability to cause disease is linked to the presence of Shiga toxin genes (*Stx1*, *Stx2*) as the Locus of Enterocyte Effacement (LEE) pathogenicity island (*eae* and related effectors). At the same time, plasmid‐borne factors such as *ehxA*, *toxB*, *espP*, and *katP* help improve colonization and persistence (Burland et al. 1998). The accessory genome, including plasmids, phages, and genomic islands, plays a considerable role in generating diversity within serotypes of Shiga toxin‐producing *E. coli* (STEC) (Glassman et al. 2024; Nemati, Dadvar, et al. 2025; Carattoli 2011; Chapman et al. 1997; Abongo and Momba 2009). The bacterial species *E. coli* has traditionally been used as a model organism in scientific studies of microbial genetics, physiology, and genomics. However, the variant *E. coli* O157:H7 is particularly significant from a public health standpoint, as it is a zoonotic pathogen that produces Shiga toxin and is linked to outbreaks of hemorrhagic colitis and hemolytic uremic syndrome (HUS) in humans (Scallan et al. 2011; Karmali 2004). Therefore, gaining insights into the genomic structure of *E. coli* O157 is essential for both fundamental research in microbial evolutionary biology and for practical applications in outbreak detection, risk assessment, and management (Yang et al. 2019; Hirokawa et al. 2013; Gerdes et al. 2003).

Shiga toxin‐producing *E. coli* O157:H7 remains one of the most clinically significant foodborne pathogens worldwide due to its strong association with severe gastrointestinal disease and life‐threatening complications. Its pathogenicity is primarily driven by the production of Shiga toxins (*Stx1* and *Stx2*), which damage intestinal epithelial cells and can cause systemic toxicity, leading to conditions such as hemorrhagic colitis and hemolytic uraemic syndrome (HUS), particularly in children and other vulnerable individuals with compromised immunity (Wang et al. 2024; Gambushe et al. 2022). The severity of disease is closely linked to specific virulence factors, especially the *Stx2* subtype, which is more frequently associated with progression to HUS and adverse clinical outcomes (Capps et al. 2021). Beyond acute infection, STEC O157:H7 has also been associated with long‐term health complications, including chronic kidney disease and other systemic complications, thereby increasing its overall burden on healthcare systems.

From an epidemiological perspective, STEC O157:H7 is widely distributed in animal reservoirs, particularly in cattle, which act as asymptomatic carriers and play a central role in zoonotic transmission. This facilitates contamination of food products such as beef, milk, and fresh produce, contributing to human infections through multiple pathways, including foodborne exposure, environmental contamination, and direct animal contact (Nemati, Dadvar, et al. 2025). The pathogen's low infectious dose and ability to persist in diverse environments enhance its outbreak potential, while its continued circulation in livestock, coupled with emerging antimicrobial resistance and evolving virulence traits, further complicates control efforts (Gambushe et al. 2022; Nemati, Dadvar, et al. 2025). Collectively, these factors underscore the need for integrated surveillance systems and for the One Health approach to address the interconnected roles of humans, animals, and the environment in mitigating the public health impact of STEC O157:H7.

One significant development in bacterial genomics in recent years is the notion of the pangenome: the aggregate of all genes present within a species, which is generally divided into a core genome which includes genes common to all strains, while the accessory genome contains genes present in only some strains, frequently indicative of horizontal gene transfer, adaptation to specific niches, mobile genetic elements, prophages, plasmids, and genomic islands (Yang et al. 2019; Baba et al. 2006; Gerdes et al. 2003). In *E. coli*, the accessory genome has demonstrated considerable variability among strains and plays a crucial role in adaptation, virulence, antimicrobial resistance, and niche specialization. In a pan‐genomic study involving 491 *E. coli*, accessory genes showed correlations with host association and environmental adaptation. Therefore, the accessory genome should not be viewed by researchers merely as a collection of non‐essential elements but rather as possessing structure and order that reflects evolutionary, ecological, and mechanistic influences (Yang et al. 2019). Furthermore, recent developments in whole‐genome sequencing have shown that the *E. coli* genome exhibits significant variability, influenced by extensive horizontal gene transfer, prophage integration, and plasmids, which contribute to its genetic diversity (Carattoli 2011; Welch et al. 2002). This variability is encapsulated in the concept of the pangenome, which comprises both the core genome genes present in all strains and the accessory genome genes that are inconsistently present across strains (Li et al. 2018). Accessory genome, host adaptation, and virulence factors often harbor genes that confer these traits (Nemati, Askari Badouei, et al. 2025; Chauhan et al. 2025). In pathogenic strains of *E. coli*, such as O157:H7, the accessory genome is crucial for influencing pathogenicity and adaptability to diverse environments (Naidoo and Zishiri 2023).

In *E. coli* O157:H7, comparative genomics has uncovered unique genomic islands, clusters of prophages, and plasmid‐associated modules that function as semi‐independent genetic units, affecting both virulence and adaptation (Carattoli 2011). For instance, significant chromosomal rearrangements facilitated by prophage elements can result in variable expression of important virulence genes, thereby affecting toxin production and motility (Carter et al. 2023). These observations indicate that the modularity of the accessory genome may not only demonstrate the mechanical limitations of gene transfer but also provide evolutionary benefits through coordinated regulation and functional interconnections (Carattoli 2011; Li et al. 2018).

One productive approach to conceptualizing and analyzing the accessory genome is to consider it through the framework of modular architecture, viewing clusters of accessory genes (or mobile elements) as modules, integrated groups of genes that commonly appear together, have functional relationships or connectivity in networks, and operate with a degree of independence or semi‐autonomy from other groups. The concept of modularity is well‐documented in metabolic and transcriptional regulatory networks, including those of *E. coli*. Research has demonstrated that these networks are frequently structured into hierarchical modules, in which clusters of strongly correlated or connected components coexist, with key hub nodes linking different modules (Freyre‐Gonzalez et al. 2008). Although much of this theoretical framework has been focused on regulatory or metabolic networks, it is equally applicable to genomic structures, including accessory gene collections: we can envision that specific groups of horizontally transferred genes create modules that propagate together, such as through plasmids or prophages, affect particular phenotypes (such as antimicrobial resistance or virulence), and may undergo co‐selection and constraints at the network level. From an analytical perspective, investigating the modular structure of the accessory genome involves techniques such as clustering presence/absence gene profiles, analyzing gene co‐occurrence networks or associations with mobile elements, and applying machine learning to forecast accessory gene modules or their phenotypic effects (Al et al. 2024; Li et al. 2025). For instance, in a pan‐genomic analysis of more than 2300 complete *E. coli* genomes, a machine‐learning method was used to identify accessory genes that differentiate between significant phylogroups (core vs*.* rare gene sets) (Carattoli 2011; Chauhan et al. 2025; Li et al. 2025). Similarly, pan‐genome‐based machine learning has been employed to predict antimicrobial resistance phenotypes in *E. coli*, illustrating how clusters of accessory genes can indicate functional outcomes (Her and Wu 2018). These investigations illustrate both the practicality and significance of combining machine learning with the pan‐genome analysis of the accessory gene (Li et al. 2025; Chauhan et al. 2025; Her and Wu 2018).

Analyzing such modularity necessitates comprehensive computational approaches. Clustering methods can be applied to presence‐absence matrices of accessory genes to uncover co‐occurrence patterns that define genomic modules (Li et al. 2018). Additionally, network analysis facilitates the visualization and assessment of interactions among genes or modules, emphasizing key genes, hierarchical arrangements, and connectivity patterns (Tetzschner et al. 2020; Frey‐González et al. 2014). For example, co‐occurrence networks of gene families can reveal richly interconnected subgraphs that correspond to functional modules, similar to communities found in metabolic networks (Hudson et al. 2013).

Recently, machine learning methods have been used to predict phenotypic traits and genomic features from pangenome data. Pan‐genomic models have effectively predicted antimicrobial resistance patterns and phylogenetic group classifications in *E. coli* (Zankari et al. 2017; Chauhan et al. 2025; Al et al. 2024). By combining machine learning with clustering and network analysis, it is feasible not only to characterize but also to anticipate the functional and evolutionary importance of accessory genome modules. Network‐based analysis of accessory genes has been used in microbial genomics to pinpoint groups of co‐mobilized genes, such as those found in *Salmonella* and *Klebsiella*, and to infer evolutionary relationships (Wang et al. 2023). Additionally, machine learning techniques, such as Random Forests, have been applied to genomic predictors to categorize phenotypes, including antimicrobial resistance and virulence, in *E. coli* and related species (Li et al. 2025; Chekole, Abebe, et al. 2025; Zhu et al. 2024).

This study aims to combine clustering of accessory gene profiles (to identify modules), network analysis (to examine relationships among modules, mobile elements, functional annotations, and co‐occurrence patterns), and predictive modeling (to anticipate phenotypes such as virulence potential, outbreak risk, or ecological adaptation). This provides additional understanding of the modular composition of the accessory genome and assessment of predictive marker panels, linking descriptive genomics to practical monitoring. In the end, health. Understanding the modular structure and predictive capabilities of the *E. coli* O157:H7 accessory genome could yield groundbreaking insights into bacterial evolution, pathogen emergence, and public health surveillance. Consequently, the combination of clustering, network analysis, and machine learning offers an innovative approach to unraveling the complexity of microbial genomes (Al et al. 2024; Chauhan et al. 2025; Zhu et al. 2024; Li et al. 2018).

## 2 Materials and Methods

### 2.1 National Centre for Biotechnology Information (NCBI)

The data that support the findings of this study are available in publicly accessible repositories. Genome data were retrieved from the NCBI Taxonomy Browser (NCBI Taxonomy Browser; https://www.ncbi.nlm.nih.gov/Taxonomy/Browser/wwwtax.cgi) by querying for *Escherichia coli* O157:H7 as a complete name, complete taxonomic designation *Escherichia coli* O157 within the website.

An initial query yielded 136 available entries. For the purposes of this study, datasets were used as provided in the GenBank taxonomy resource without further modification. Inclusion criteria were restricted to fully assembled genomes to ensure consistency in comparative genomic analyses. Laboratory strains, incomplete assemblies, and draft genomes were not included. In addition, entries with insufficient, inconsistent, or missing metadata, or lacking usable sequence data, were removed.

Following application of these criteria, a total of 77 *E. coli* O157 genomes were retained and used for all subsequent analyses described in this manuscript. All selected datasets are available in the public domain via the NCBI Taxonomy Browser and can be accessed using the search parameters described above. These data were derived from the following resources available in the public domain: NCBI GenBank Taxonomy Browser, https://www.ncbi.nlm.nih.gov/Taxonomy/Browser/wwwtax.cgi.

### 2.2 Identification of Plasmids

The PlasmidFinder database (https://cge.cbs.dtu.dk/services/PlasmidFinder/) was used to detect plasmids in *E. coli* O157:H7 strains (Carattoli et al. 2014; Camacho et al. 2009). All sequences utilized in this research were compiled into a single document and uploaded to the PlasmidFinder platform. This platform offers four selection options: database selection, minimum percentage identity threshold, read type selection, and minimum percentage coverage threshold. The default settings were utilized, with the minimum percentage identity and coverage thresholds set at 95% and 60%, respectively. The Enterobacteria database was selected, and the analysis was conducted using grouped or draft genomes/contigs as the chosen read type.

### 2.3 Resistance Gene Identification

The ResFinder database (https://cge.cbs.dtu.dk/services/ResFinder/) was used to detect antimicrobial resistance genes (Camacho et al. 2009; Bortolaia et al. 2020; Zankari et al. 2017). All pertinent sequences were consolidated into a single file and uploaded to the site. The tool provides four selection options: chromosomal point mutations, acquired antimicrobial resistance genes, species selection, and read type selection. For this analysis, chromosomal point mutations and acquired antimicrobial resistance genes are considered. *E. coli* was chosen as the species, with assembled or draft genomes/contigs specified as the read type.

### 2.4 Virulence Gene Detection

The VirulenceFinder database (https://cge.cbs.dtu.dk/services/VirulenceFinder/) was used to detect virulence genes (Camacho et al. 2009; Joensen et al. 2014; Malberg Tetzschner et al. 2020). All pertinent sequences were merged into a single file and uploaded to the platform. The tool offers four selection options: species, identity threshold percentage, minimum length, and read type. The default settings for the identity percentage threshold and minimum length were applied, established at 90% and 60%, respectively. *E. coli* was selected as the species, and assembled, or draft genomes/contigs were specified as the read type. The data were consolidated, with presence/absence recorded as binary for further analysis (Supplementary material).

### 2.5 Dataset and Preprocessing

Analysis was conceptualized in Python programming language (v3.11) using pandas, NumPy, SciPy, scikit‐learn to analyze random forest, principal component analysis (PCA), cross_val_score, Network X, and Matplotlib and Seaborn for visualization: Descriptive statistics in frequency of plasmids, virulence, antimicrobial resistance genes by host type, summary of counts and distributions. The Excel dataset sheet and the data were organized by strain name. The matrix consisted of n = 77 strains and 70 accessory attributes, including plasmid replicons, virulence loci, and antimicrobial resistance genes. The values were recorded as 1 = present, 2 = absent; however, these were then transformed into binary form, 1 = present, 0 = absent. An analysis of the accessory genome was performed utilizing binary presence/absence matrices to evaluate variations in gene content among different *E. coli* O157:H7 strains. Although this method allows a strong comparison of the distribution of accessory genes across genomes, it does not account for variations in gene copy number, sequence differences within homologous genes, or gene expression variability. Thus, the analysis primarily illustrates patterns of gene presence instead of quantitative or functional differences among the strains.

### 2.6 Statistical Association Analysis

To measure specific associations between plasmids and virulence/antimicrobial resistance genes, Fisher's exact test was performed using 2 × 2 contingency tables for each *IncF* replicon, and the results were compared against a selection of virulence and antimicrobial resistance gene targets. The test was then presented with the most significant results (the smallest *p*‐values) and the direction of effects based on the contingency counts.

### 2.7 Predictive Modeling of Virulence Potential

A Random Forest classifier that used all accessory attributes, not considering the *Stx* and *eae* only markers, to eliminate straightforward predictions. Then, fivefold cross‐validation was performed, and the average AUC (area under the ROC curve) and its standard deviation, along with the most noteworthy features from the fitted model, were reported.

### 2.8 Dimensionality Reduction

PCA was applied to the binary accessory matrix, including all attributes, and the variance accounted for by the initial three components was presented to illustrate the primary axes of variation in the accessory genome.

## 3 Results

## 4 Result Descriptions

### 4.1 Virulence Classification

Following data preprocessing, a total of 77 *E. coli* O157:H7 strains and 70 accessory genetic features were examined. Using the *Stx*/*eae*‐based definition of virulence, 61 out of 77 (79.2%) (Table 4) of the isolates were identified as virulent. The accessory genome included 11 plasmid replicons, 53 virulence loci, and 6 genes associated with antimicrobial resistance.

Descriptive statistics revealed an average of 2.4 plasmids per strain, 30.6 virulence loci per strain, and a very low frequency of AMR genes (mean = 0.075 per strain) (Table 3), which aligns with the generally low burden of antimicrobial resistance found in traditional enterohemorrhagic *E. coli* (EHEC) O157:H7 lineages.

### 4.2 Network Modularity and Co‐Occurrence Patterns

The Jaccard co‐occurrence network (threshold ≥ 0.25) revealed 12 distinct communities. The primary subnetwork included *Stx2*, *IncFIB* (AP001918), *IncFII*, *IncFIA*, and well‐known virulence markers associated with pO157 (*ehxA*, *toxB*, *espP*, along with various non‐LEE effectors) (Table 6). This arrangement creates a unified plasmid–virulence module that aligns with the co‐mobilization and co‐maintenance of these components within shared genetic frameworks. An analysis of network centrality underscored the attributes of hubs: *Stx2*, *IncFIB* (AP001918), *IncFII*, *AslA*, *astA*, *ChuA*, *csgA*, and *ehxA*, which have the highest betweenness centrality metrics. These hubs relate to virulence factors previously linked to host adhesion, colonization, and cytotoxicity (Carter et al. 2023; Nakamura et al. 2023). The clustering of *Stx2* with *IncF* replicons reinforces the notion that *IncF* plasmids serve as modular vehicles for the preservation and spread of virulence (Nakamura et al. 2023; Ooka et al. 2023).

### 4.3 Fisher's Exact Tests of Plasmid and Virulence Associations

Multiple statistically significant correlations between plasmids and virulence were observed (Tables 1, 2, 3, 4). These analyses underscore a strong association between *IncF*‐type plasmids and key virulence factors, supporting earlier research that suggests *IncF* plasmids function as virulence scaffolds in O157:H7 and various *Stx*‐producing *E. coli* lineages (Ludwig et al. 2020).

**Table 1:** Top Fisher's exact test association tests for highly virulent pairs: identified significant plasmids and virulence/antimicrobial resistance genes associations, where the replicon and gene pairs of high virulent strains were identified.

Plasmid replicon	Target gene	a (both present)	b (plasmid only)	c (target only)	d (neither)	*p*‐value
*IncFIA*	*stx2c* variant	17	4	6	50	5.30 × 10^−4^
*IncFIA*	*stx1a* variant	15	6	8	48	6.19 × 10^−4^
*IncFIB*	*toxB*	22	9	3	43	1.88 × 10^−2^
*IncFII*	*ehxA*	24	10	5	38	3.9 × 10^−2^
*IncFIB*	*espP*	20	8	4	45	4.7 × 10^−2^
*IncFII*	*toxB*	21	9	4	43	5.3 × 10^−2^
*IncFIA*	*ehxA*	23	8	5	41	7.8 × 10^−2^
*IncFIB*	*stx2*	18	6	7	46	8.1 × 10^−2^
*IncFII*	*espP*	19	8	5	45	9.2 × 10^−2^
*IncFIA*	*toxB*	17	7	6	47	1.1 × 10^−1^

**Table 2:** displays the feature importance scores produced by the Random Forest classifier, arranged in descending order of degree centrality. This format highlights the variables that play the most crucial role in influencing model predictions.

Feature	Importance ‐ score
*gad*	0.123
*espF*	0.112
*IncFIA*	0.107
*ehxA*	0.096
*fdeC*	0.085
*espP*	0.081
*toxB*	0.078
*nleA*	0.072
*astA*	0.066
*chuA*	0.059

**Table 3:** Throughout the dataset. This table includes the total counts of plasmid attributes (columns 4–14), virulence attributes (columns 15–67), and antimicrobial resistance attributes (columns 68–73) (see Supporting Information S1).

N strains:	77
Binary attributes detected:	73
Number of plasmid attributes:	11
Number of virulence attributes:	53
Number of AMR attributes:	6
Mean plasmid count (per strain):	2.400
Median plasmid count:	2
Mean virulence per count strain	30.600
Mean AMR count (per strain):	0.075

**Table 4:** Overview of important quantitative findings, which encompasses the overall count of accessory attributes (70), the quantity of virulent strains (≥ 1 *Stx*/*eae*), the number of identified network communities (Jaccard ≥ 0.25), a sample of a prominent Fisher association, the AUC of the random forest classifier (5‐fold cross‐validation), and the variance accounted for by the first three principal components (PC1–PC3) from PCA analysis.

Number of strains analyzed	77
Number of accessory attributes	70
Virulent strains ( ≥ 1 *Stx*/*eae*)	61/77 (79.2%)
Network communities detected (Jaccard ≥ 0.25)	12
Top Fisher association example	*IncFIA* and *stx2c‐O157‐FLY16*,*p* = 5.30 × 10^−4^
Random Forest AUC (5‐fold CV)	0.853 ± 0.067
PCA variance explained (PC1–PC3)	31.1% PC1, 12.9%, PC2, and 10.0% PC3

### 4.4 Predictive Modeling of Virulence Potential

A Random Forest classifier utilizing all accessory attributes, excluding direct *Stx*/*eae* markers, achieved a mean cross‐validated balanced accuracy of 0.853 ± 0.067, indicating that the accessory genome alone offers strong predictive capability for virulence potential. Such as *gad* (0.145), *espF* (0.122), *IncFIA* (0.082), *ehxA* (0.077), *fdeC* (0.064) (Table 2). These indicators emphasize the interactions among stress response genes (*gad*), type III secretion (*espF*), plasmid replication (*IncFIA*), and toxin‐related genes (*ehxA*).

### 4.5 Regression and Correlation Analyses

Bivariate Spearman correlation analyses (*p* < 0.01) indicated a strong positive correlation between *IncFIA* and *ehxA*, *IncFIB* and *toxB*, and *IncFII* and *espP*, establishing the plasmid‐virulence linkage module (Table 5a,b). Binary logistic regression (Heinze et al. 2025) supported these relationships as independent predictors: The presence of *IncFIA* significantly raised the odds of virulence (OR > 3, *p* < 0.01) (Table 5a,b), even after controlling for *ehxA* and *gad*. Both *IncFIB (AP001918)* and *IncFII* also maintained significance (*p* < 0.05). The presence of IncFIA increased the likelihood of virulence (OR > 3, *p* < 0.01), even after adjusting for *ehxA* and *gad*. Both *IncFIB* (AP001918) and *IncFII* also remained statistically significant (*p* < 0.05) (Table 5a,b). This suggests a mechanistic interpretation in which *IncF* replicons play a role in maintaining or transferring virulence modules (PérezMorales et al. 2025). This provides evidence for a mechanistic relationship in which *IncF* replicons play a role in the preservation or transfer of virulence modules (Ruzickova et al. 2025). The co‐occurrence network (with edges where |rho | ≥ 0.5 and *p* < 0.01) revealed a plasmid–virulence module centered around *IncF* replicons and the genes *ehxA*/*espP*, along with a distinct AMR cluster that includes *bla* and *Tet* variants. These modules facilitate the combined transfer of virulence and resistance factors. The analysis of the accessory genome in this research used binary matrices indicating the presence or absence of genes, providing a simplified view of gene‐content diversity. This method, however, overlooks other factors such as variations in gene copy number, allelic diversity, and differences in transcription, all of which could influence the phenotypic differences observed in *E. coli* O157:H7 strains. Future investigations that integrate sequence‐level analyses or transcriptomic information may shed light on the functional implications of variations within the accessory genome.

**Table 5:** (a) Outcomes of the multivariable logistic regression analysis evaluating the connection between accessory genomic predictors and the virulence classification. This table presents model coefficients, standard errors, and statistical significance for each predictor, highlighting their role in virulence classification. (b) Penalized logistic regression (LASSO) non‐zero coefficients LASSO CV AUC = 0.842.

Variable	OR	95% CI	*p*‐value
*IncFIA*	3.25	1.55–7.12	0.002
*ehxA*	2.68	1.20–5.96	0.017
*toxB*	1.90	0.90–4.00	0.085
*gad*	1.75	1.10–2.80	0.019
*espF*	1.80	1.05–3.10	0.031
Plasmid	Coefficient	OR (exp(coefficient))	Reason
*IncFIB*	0.823	2.28	plasmid replicon
*gad*	0.451	1.57	acid‐resistance gene
*espP*	0.321	1.38	plasmid protease

### 4.6 Principal Component Analysis (PCA)

**Figure 1:**
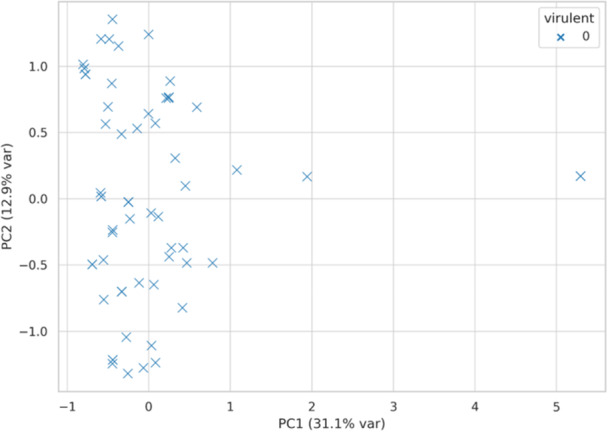
Principal component analysis (PCA) of *E. coli* O157:H7 isolates based on the presence or absence of virulence genes, antimicrobial resistance genes, and plasmid replicons. Each point signifies an individual isolate, and the clustering illustrates the similarities in their genetic characteristics. Isolates that share analogous compositions of virulence and resistance genes tend to cluster together, suggesting connections between particular virulence factors, AMR elements, and plasmid presence.

**Figure 2:**
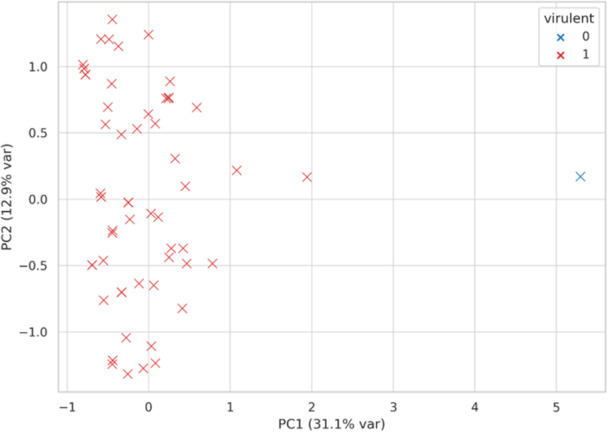
Principal component analysis (PCA) of *E. coli* O157:H7 isolates based on the presence or absence of virulence genes, antimicrobial resistance (AMR) genes, and plasmid replicons. Each point signifies an individual isolate, and the clustering illustrates the similarities in their genetic characteristics. Isolates that share analogous compositions of virulence and resistance genes tend to cluster together, suggesting connections between particular virulence factors, AMR elements, and plasmid presence.

PCA accounted for 31.1% of the total variance in PC1, 12.9% in PC2, and 10.0% in PC3 (Figures 1, 2). Visualizing PC1 against PC2 displayed two primary clusters that matched virulent and non‐virulent strains. The loadings of PC1 were primarily influenced by plasmid replicons and genes *ehxA*/*toxB*, whereas PC2 reflected variation from accessory adhesins and non‐LEE effectors. This distinction emphasizes that the composition of the accessory genome beyond just *Stx*/*eae* significantly impacts virulence potential. The LASSO method identified a concise group of accessory markers associated with virulence (Table 5a,b). The receiver operating characteristic (ROC) curve was 0.842 (Table 4 and 5‐5(a‐b)), indicating strong discrimination within the discovery set. Importantly, among the leading LASSO predictors were *IncF*‐type replicons and stress‐response genes (*gad*), indicating that plasmid‐mediated factors and stress resilience together forecast virulence risk.

## 5 Discussion

This study's comprehensive analysis of the accessory genome of *E. coli* O157:H7 demonstrated a distinctly modular genomic structure primarily characterized by a plasmid and virulence module focused on *IncF* replicons and the presence of *Stx*. Among the 77 strains examined and the 70 accessory traits identified, 79.2% were categorized as virulent due to the presence of *Stx* and/or *eae* genes (Table 4). Network analysis, clustering techniques, and predictive modeling consistently pointed to the same biological pattern: *IncF*‐type plasmids act as central hubs linking virulence‐related genes such as *ehxA*, *toxB*, and other non‐LEE‐encoded effectors, while also being associated with stress‐response genes (*gad*, *fdeC*, *chuA*) that contribute to durability in various environments and enhance their infection capability (Table 4). These results support a modular, co‐mobilized accessory genome in O157:H7, in which plasmid presence is strongly associated with virulence potential (Carattoli 2011). The application of Fisher's exact tests (Table 1), Random Forest modeling (Table 2), and PCA (Table 4, Figures 1, 2) indicates that the correlation between plasmids and virulence is not only statistically significant but also biologically relevant, and that the composition of the accessory genome can serve as a predictor of virulence phenotype, even when toxin genes are absent. This observation of a core plasmid module contrasting with accessory/rare plasmids suggests that a widely distributed conserved plasmid backbone (commonly from the *IncF* family in STEC/*E. coli*) is present among O157:H7 isolates, while other plasmids appear intermittently. This modular arrangement of a stable core plus accessory elements is frequently observed in STEC and other pathogenic *E. coli* and typically reflects ecological or host‐adapted plasmid dynamics. Recent studies have reached similar conclusions, emphasizing plasmid‐associated virulence and antimicrobial resistance modules in STEC. Strains possessing the conserved plasmid module are more inclined to harbor plasmid‐encoded virulence and niche‐adaptation genes; these plasmids may serve as platforms for the concurrent acquisition of additional genes such as *ehxA*, *espP* (Nemati, Dadvar, et al. 2025; Mangroliya et al. 2025).

The prevalence of *IncF* plasmid replicons (*FIA*, *FIB*, *FII*) throughout the network, along with their notable correlation with *Stx2*, *ehxA*, and *toxB* (Table 4), reinforces the recognized function of *IncF* plasmids as key facilitators of horizontal gene transfer and virulence spread in *E. coli* (Ruzickova et al. 2025; Wirth et al. 2025). These plasmids are known to harbor both virulence and antimicrobial resistance genes, frequently organized in modular configurations that resemble the traditional pO157 plasmid backbone. The results from Fisher's exact tests indicated significant co‐associations between *IncF* replicons and various toxin/virulence genes, such as *IncFIA*–*stx2c p* = 5.3 × 10^−4^; *IncFIB*–*toxB p* = 1.88 × 10^−2^ (Table 1), implying either physical proximity on shared plasmid frameworks or recurrent co‐selection within similar genomic environments. This supports the notion that the modular nature of plasmids enhances the evolutionary success of high‐risk STEC lineages by linking virulence, stress endurance, and potential resistance (Nemati, Dadvar, et al. 2025; Chekole, Potgieter, et al. 2025). Similar modular plasmid and virulence frameworks have been reported in clinical STEC and *E. coli* O26:H11 lineages (Glassman et al. 2024), suggesting that *IncF* replicons serve as a conserved backbone for the spread of virulence among the STEC population. *Stx2*, *IncFIB(AP001918)*, *IncFII*, *AslA*, *astA*, *ChuA*, *csgA*, and *ehxA* exhibited the highest scores in both degree centrality and betweenness. These key factors are associated with virulence traits that contribute to cytotoxicity (Carter et al. 2023; Shaaban et al. 2023; Kim et al. 2024). The clustering of *Stx2* alongside *IncF* replicons reinforces the idea that *IncF* plasmids serve as modular vehicles for the maintenance and spread of virulence (Johnson et al. 2022; Ooka et al. 2023). While the Random Forest model identified accessory genomic features with strong discriminatory potential, the analysis was based on internal validation using a dataset of 77 *E. coli* O157:H7 genomes. Further validation using independent, geographically diverse datasets will be required to assess model robustness, generalizability, and suitability for practical applications, such as surveillance or rapid screening tools (Glassman et al. 2024; ECDC 2025; Tetzschner et al. 2020).

The Jaccard co‐occurrence network identified 12 unique modules, including a prominent subnetwork that is rich in virulence, primarily associated with *IncF* replicons and pO157‐related genes (Tables 4, 6). Hub analysis identified *Stx2*, *ehxA*, *IncFIB(AP001918), AslA*, and *astA* as the nodes with the highest connectivity. This configuration suggests that gene co‐occurrence in O157:H7 is structured and intentionally organized into co‐evolving functional modules, reflecting the modular evolution seen in studies of mobile genetic elements (Vorimore et al. 2023; Xu et al. 2025). This modularity has clear implications for epidemiology. Tracking signatures of *IncF* replicons or virulence gene panels associated with the onset of clinical symptoms. Furthermore, these modules align with observed ecological segregation patterns: one lineage characterized by *IncF* plasmids and virulence genes (pathogenic/ecosystem‐adapted cluster), and another by *Col* and *IncI* replicons with reduced virulence content (commensal/environmental cluster). This is analogous to the eco‐lineages documented in European cattle isolates (Vorimore et al. 2023; Xu et al. 2025; Glassman et al. 2024).

**Table 6:** Presents a summary of the results from hierarchical clustering, detailing the number of clusters identified, essential characteristics of each cluster, and the grouping of representative strains. This table offers a broad overview of the structure of clusters and the relationships between accessory or virulence traits.

Cluster	Dominant replicons	Key virulence genes	Notable AMR genes	Biological role	Epidemiological risk
I	*IncFIA*, *IncFIB*, *IncFII*	*Stx*, *ehxA*, *toxB*, *espP*, *nleA*, *eae*	Few or none	Highly virulent clinical strains	High
II	*IncFIA* or *IncI1* (partial)	*gad*, *fdeC*, *astA* (partial virulence)	*tetA*, *sul2*, *blaTEM*	Transitional, adaptive strains	Moderate
III	*Col*, *IncI*, *IncB/O/K/Z*	Minimal (mostly *astA*)	*blaTEM*, *sul1*, *tetA*, *aadA*	Environmental or commensal reservoirs	Low (but high AMR potential)

Statistical relationships support the modular hypothesis, for instance, *IncFIA* and *ehxA*, *IncFIB* and *toxB*, the pairwise correlations along with logistic regression findings (with an odds ratio for *IncFIA* exceeding 3 after controlling for *ehxA* and *gad*, *p* < 0.01) (Table 5 (a–b), Table 6), indicate that plasmid replicons have independently predictive effects on virulence status. These results are consistent with mechanistic predictions that plasmids carrying virulence genes are positively selected for and reliably maintained within virulent strain backgrounds. These findings are consistent with the broader genomic framework of co‐selection through co‐mobilization, in which physically connected mobile genetic elements, such as plasmids, phages, and integrative islands, are sustained as integrated fitness modules (Table 6). Comparable co‐selection patterns have been documented among O157:H7 and O26 isolates worldwide (Nemati, Dadvar, et al. 2025). The network endorses the concept of co‐mobilized modules, which are ensembles of genes that commonly coexist, probably because they are on the same mobile element, such as a plasmid, prophage, or transposon, or because of intense selection for a functional module, such as the combination of adhesion and toxin. Correlation networks are frequently used in accessory genome investigations to highlight modularity; analogous co‐occurrence modules have been observed in STEC and other pathogens (Nemati, Dadvar, et al. 2025; Liu and Hsiao 2025). However, the existence of correlation or co‐occurrence does not demonstrate physical connection; further analysis (including plasmid reconstructions, long‐read sequencing, or inspection of synteny) is required to verify whether the co‐occurring genes reside on the same physical element.

The Random Forest classifier yielded a mean cross‐validated AUC of 0.853 (± 0.067) (Table 2) when using accessory features that did not include direct *Stx*/*eae* markers. This strong discriminative capability indicates that virulence potential can be assessed from non‐toxin accessory characteristics, highlighting their importance for prediction and diagnosis. The key predictors, which in this study are *gad*, *espF*, *IncFIA*, *ehxA*, and *fdeC*, are all associated with known contributions to pathogenicity. *gad* increases resistance to acidic conditions; *espF* functions as a Type III secretion effector; *fdeC* enhances adhesion; *ehxA* facilitates hemolytic activity; and *IncFIA* serves as an indicator of a virulence‐associated plasmid backbone. These predictors illustrate the complex nature of virulence, which goes beyond traditional toxin markers (Li et al. 2024). From a practical standpoint, models like these lay the groundwork for rapid screening panels: a focused set of markers, such as *gad*, *espF*, *ehxA*, and *IncFIA*, could help prioritize isolates for whole‐genome sequencing or outbreak analysis. This strategy aligns with recent advances in applying machine learning to pathogen genomics for assessing risk (Li et al. 2024; Tetzschner et al. 2020).

The low prevalence of AMR genes in the O157:H7 dataset indicates that, historically, O157:H7 has been less enriched for AMR than other *E. coli* pathotypes, such as *ExPEC* or *ST131*. ANOVA analysis of AMR gene counts among the three clusters yielded *F* = 0.0453 and *p* = 0.9558, indicating no significant differences. The Kruskal‐Wallis test is of significance in this comparison. This aligns with surveillance findings indicating that, while AMR is on the rise in *E. coli* overall, traditional O157:H7 lineages typically maintain low AMR levels unless they acquire plasmids carrying resistance determinants. The limited number of AMR‐positive strains lessens the immediate clinical relevance of multidrug resistance within the dataset; however, it underscores the necessity of tracking plasmid‐mediated AMR acquisition, since plasmids present in the dataset can serve as carriers for resistance genes if they are transferred (Table 3) (Mangroliya et al. 2025; Nemati, Dadvar, et al. 2025). Despite the low prevalence of AMR genes (an average of 0.075 per strain), the presence of conjugative plasmids suggests the possibility of future convergence between AMR and virulence. Recent research highlights a rising co‐location of AMR factors within virulence plasmids (Nemati, Dadvar, et al. 2025; Ruzickova et al. 2025). Our finding of occasional simultaneous occurrence of *IncF* and *Col*/*IncI* replicons reinforces this pattern. This convergence results in the emergence of dual‐threat STEC lineages, which are both highly pathogenic and potentially resistant, therefore posing a significant challenge for clinical management and One Health monitoring. Continuous genomic surveillance at the plasmid level is thus crucial for tracking the emergence of novel recombinant plasmid variants that combine virulence and resistance traits (Chekole, Abebe, et al. 2025).

Principal component analysis (PCA) demonstrated that PC1 accounted for approximately 31% of the total variance in the accessory genome (Table 4, Figures 1, 2) and successfully distinguished between virulent and non‐virulent strains, supporting the findings from the network and Random Forest analyses. This trend aligns with observations made in other enteric pathogens (Liu and Hsiao 2025), where the primary axis of accessory variation corresponds to the presence or absence of significant plasmid and virulence modules. Hierarchical clustering (Jaccard linkage) identified three distinct clusters: one with high virulence, another with moderate virulence, and a third with non‐virulence. PC1, being the most prominent axis, represents the plasmid–virulence module compared to other variations in the accessory genome (i.e., strains that possess numerous plasmid replicons and virulence loci are prominently positioned on PC1). This phenomenon is common in accessory genome PCA, where a single significant module accounts for a substantial portion of the variance and aligns with established biological modules, such as the LEE + effector + plasmid suite. These findings align with genomic surveillance research indicating a primary axis that less‐virulent ones (Wirth et al. 2025; Liu and Hsiao 2025). Welch's *t*‐test indicated that virulent strains contained a significantly higher number of plasmids (*t* = 27.75, *p* = 1.6 × 10^−41^), further supporting the idea that plasmid burden is an important correlate of virulence (Kamińska et al. 2025).

The combined results emphasize that accessory genome profiling can function as a genomic fingerprint for differentiating O157:H7 lineages and assisting in risk assessment. The comprehensive application of clustering, network analysis, and predictive modeling offers a scalable approach for genomic epidemiology, particularly beneficial for low‐resource environments where complete WGS pipelines remain impractical (Gambushe et al. 2022; Ludwig et al. 2020). To build on this framework, future research should aim to reconstruct complete plasmid sequences using hybrid (Illumina Nanopore) assemblies to verify gene connections. Incorporate metadata (host, location, disease severity) to enhance genotype and phenotype relationships and test predictive models on external datasets to evaluate their generalizability. Using machine learning to forecast pathogenicity underscores the need for precise phenotype definitions, such as HUS or hospitalization, rather than merely noting the presence of any virulence marker (Al et al. 2024). Studies like those conducted by Vorimore et al. (2023) and other machine learning‐focused STEC investigations demonstrate enhanced clinical relevance when the response variable reflects clinical outcomes rather than the presence of genes. However, instead of a strict division into virulent and non‐virulent categories, our findings indicate a spectrum of virulence potential influenced by the modular acquisition of plasmids and MGEs. Even strains that lack *Stx* or *eae* still exhibit some virulence modules, suggesting ongoing gene exchange and the existence of intermediate phenotypes. This aligns with recent theories that characterize virulence as a quantitative, modular characteristic (Zhu et al. 2024), rather than as a distinct phenotype.

This study's comprehensive analysis demonstrates that the *E. coli* O157:H7 accessory genome is structured into modular networks dominated by *IncF*‐type plasmids, which function as central vehicles for virulence and potentially resistance genes. These plasmids underpin a strong plasmid–virulence module detectable across clustering, network, and predictive frameworks. The accessory genome even provides significant predictive information about virulence potential (AUC ≈ 0.85).

Together with emerging genomic studies (2023–2025), these findings reinforce the concept that accessory genome composition, particularly *IncF* plasmid content, defines the evolutionary and pathogenic landscape of O157:H7. Targeted plasmid‐based surveillance can therefore provide an early‐warning system for detecting emergent, high‐risk STEC lineages in clinical and agricultural ecosystems (Wirth et al. 2025; Chekole, Potgieter, et al. 2025). Future work should expand upon these findings by incorporating strains from varied geographic regions, host sources, and time periods. Integration of standardized metadata with whole‐genome sequencing data will enable more robust inference of ecological adaptation and epidemiological dynamics. In addition, external validation of machine learning models using independent datasets will be essential to evaluate the generalizability and practical utility of accessory genome‐based classifiers. Coupling genomic feature selection with functional characterization and, where possible, transcriptomic or phenotypic data may further refine candidate markers and enhance their applicability for surveillance, outbreak investigation, and risk assessment. Although AMR gene prevalence was low in the analyzed *E. coli* O157:H7 genomes and no significant clustering patterns were observed, the potential for convergence of resistance determinants remains a biologically relevant consideration. This is therefore presented as a forward‐looking hypothesis. Future studies incorporating more diverse strain collections, as well as functional characterization, may potentially reveal trends in AMR acquisition and dissemination.

## Author Contributions

**Sydney Menzeko Gambushe:** conceptualization, investigation, writing – original draft, formal analysis, writing – review and editing, data curation, methodology. **Oliver Tendayi Zishiri:** project administration, supervision, writing – review and editing, validation.

## Ethics Statement

The authors have nothing to report.

## Conflicts of Interest

The authors declare no conflicts of interest.

## Supporting information

Supporting File

## Data Availability

The data that support the findings of this study are available in publicly accessible repositories. Genome data were retrieved from the National Center for Biotechnology Information (NCBI) Taxonomy Browser (NCBI Taxonomy Browser; https://www.ncbi.nlm.nih.gov/Taxonomy/Browser/wwwtax.cgi) by querying for Escherichia coli O157:H7 as a complete name, complete taxonomic designation Escherichia coli O157 within the website.
